# Neoadjuvant Therapy in Operable Breast Cancer: Application to Triple Negative Breast Cancer

**DOI:** 10.1155/2013/219869

**Published:** 2013-08-01

**Authors:** Foluso O. Ademuyiwa, Matthew J. Ellis, Cynthia X. Ma

**Affiliations:** Section of Breast Oncology, Division of Oncology, Department of Medicine, Siteman Cancer Center, Washington University School of Medicine, 660 South Euclid Avenue, St. Louis, MO 63110, USA

## Abstract

Systemic treatment for triple negative breast cancer (TNBC: negative for the expression of estrogen receptor and progesterone receptor and HER2 amplification) has been limited to chemotherapy options. Neoadjuvant chemotherapy induces tumor shrinkage and improves the surgical outcomes of patients with locally advanced disease and also identifies those at high risk of disease relapse despite today's standard of care. By using pathologic complete response as a surrogate endpoint, novel treatment strategies can be efficiently assessed. Tissue analysis in the neoadjuvant setting is also an important research tool for the identification of chemotherapy resistance mechanisms and new therapeutic targets. In this paper, we review data on completed and ongoing neoadjuvant clinical trials in patients with TNBC and discuss treatment controversies that face clinicians and researchers when neoadjuvant chemotherapy is employed.

## 1. Introduction

Neoadjuvant chemotherapy, also known as preoperative or primary systemic therapy, is an option for patients with breast cancers who require cytotoxic chemotherapy. It was initially used for patients with locally advanced inoperable breast cancers [1]. Subsequently tumor regression induced by chemotherapy allows a proportion of patients with large operable cancers who hitherto required a mastectomy to achieve breast conservation. For example, the National Surgical Adjuvant Breast and Bowel Project (NSABP) B-18 clinical trial randomized a large number of women to receive chemotherapy either pre- or postoperatively [2]. Although there were no survival differences, preoperative chemotherapy improved the rate of breast conservation. In those who are already candidates for breast conservation, neoadjuvant chemotherapy may also result in a more desirable cosmetic outcome by allowing less extensive surgery. In addition, neoadjuvant treatment provides a critical opportunity to assess the in vivo responsiveness to chemotherapy and a research platform for investigations of tissue or imaging predictors of response and novel therapeutic targets. Neoadjuvant chemotherapy has therefore increasingly become a preferred strategy for patients with Stage II or III breast cancers. 

Triple negative breast cancer (TNBC) is defined clinically by the absence of estrogen receptor (ER), progesterone receptor (PgR), and HER2/neu overexpression and encompasses a molecularly diverse group of diseases. As TNBC lacks a clearly defined therapeutic target, patients receive chemotherapy for their systemic management. Since chemotherapy-resistant TNBC carries a particularly poor prognosis, the identification of the mechanism of chemoresistance and therapeutic advances are critical. There is therefore a particularly strong rationale for clinical research in this setting.

## 2. Molecular Classification of TNBC

Breast cancer is subdivided into at least five major intrinsic subtypes, including luminal A, luminal B, HER2-enriched, basal-like, and claudin-low breast cancer [3]. The majority of TNBCs belong to the basal-like breast cancer (BLBC) subtype, which is characterized by basal gene signature containing keratins 5, 6, and 17 and high expression of proliferation-related genes [4]. TNBC and BLBC do not completely overlap, and the concordance rate ranges from 70 to 90% in various studies [5, 6]. The other less commonly defined subtypes of TNBC include (confusingly) luminal tumors. The Cancer Genome Atlas (TCGA) analysis along with a number of other genomic studies [7–12] have demonstrated that BLBC is characterized by frequent alterations in the TP53 pathway, PIK3CA/PTEN pathway, and RB1 pathway and a high frequency of aneuploidy, genomic instability, and Myc amplification (Table 1). Interestingly, the genomic alterations of BLBC, including BRCA1 inactivation, RB1 loss, cyclin E1 amplification, high expression of AKT3, MYC amplification/high expression, a high frequency of TP53 mutations, and high pathway activity of the HIF1-a/ARNT, MYC, and FOXM1 regulatory hubs, resemble those of serous ovarian carcinoma, indicating potential similarities between the two cancer types in regards to pathogenesis and therapeutic opportunities [7]. Incorporation of molecular understanding to drug development is undoubtedly a major research focus in breast cancer in the years to come [13].

In an effort to identify TNBC-specific molecular subtypes, Lehmann et al. analyzed the gene expression profile of 587 TNBC cases [14]. Six subtypes were identified, including the two basal-like subtypes, BL1 and BL2, which were the most prevalent and were so named because of their similarity to the basal-like intrinsic subtype. These tumors have high expression of genes involved in cell cycle and division and are highly proliferative as marked by high Ki-67 staining. These results suggest that chemotherapies that target cell division and mitosis may be most appropriate in this class. In a recent retrospective study, BL1 and BL2 subtypes were associated with a higher rate of pCR (63%; *P* = 0.042) with third generation, taxane containing regimens, as compared to mesenchymal-like (31%) or luminal androgen receptor (14%) subtypes [15]. In addition, elevated expression of DNA damage response pathway genes was present in the BL1 subtype, and representative cell lines were found to be preferentially responsive to cisplatin which induces DNA damage through the formation of predominantly guanine cross-links. A third subtype, “immunomodulatory,” was found to be enriched in genes involved in immune processes. These include immune transduction pathways, cytokine signaling such as IL-2 pathway, and antigen processing, among others. This subtype may represent medullary breast cancer, a subtype of TNBC that has a relatively good prognosis, based on a similar expression profile reported in another study [16]. Mesenchymal (M) and mesenchymal stem-like (MSL) subtypes were characterized by expression of cell motility genes and proteins of the extracellular matrix. The MSL subtype displayed low expression of claudins 3, 4, and 7 and therefore overlaps with the claudin-low subtype of breast cancer. The MSL subtype also expressed genes involved in growth factor signaling such as EGFR and PDGFR, pointing to possible therapeutic options in this subtype. The sixth subtype, luminal androgen receptor (LAR), was found to be enriched in genes involved in steroid synthesis and androgen metabolism. It has been reported previously that a proportion of TNBC may have a steroid hormone gene regulation signature despite being negative for ER and PR [17]. This finding was replicated in the study by Lehmann et al. Androgen receptor mRNA was expressed at an average of ninefold higher level in this subtype than all the other subtypes [18]. Interestingly, LAR subtype is classified to either luminal A or luminal B intrinsic subtype despite being negative for ER expression. The finding of LAR subtype presents a potential venue for endocrine treatment for at least a proportion of TNBC patients and clinical trials are underway. 

It is likely that the classification of TNBC will continue to evolve and we envision the development of treatment-directed classification to also include necessary markers by either genomic or proteomic analysis. Neoadjuvant setting provides a platform for proof of concept studies in this regard. A consensus conference on TNBC definitions, similar to historic efforts in lymphoma and leukemia, should be considered to facilitate subset specific clinical trials.

## 3. Neoadjuvant Chemotherapy for TNBC

 While several clinical trials involving traditional chemotherapy, or biologics have recently been launched in an attempt to improve the outcome of patients with TNBC, most neoadjuvant studies have not restricted entry criteria to only patients with TNBC and so the attempt to define treatment standards has required retrospective subset analysis, risking underpowered investigation. Nonetheless a large proportion (41%) of the study population in NSABP protocol B-40 was classified as having TNBC [19]. The goals of this study were to determine if the addition of capecitabine or gemcitabine to docetaxel followed by doxorubicin and cyclophosphamide (AC) would increase pathologic complete response (pCR) rates in patients with palpable and operable HER2-negative disease and also to determine whether the addition of bevacizumab to docetaxel-based regimens followed by AC will increase pCR rates. In the chemotherapy alone arms, the addition of capecitabine or gemcitabine to docetaxel versus docetaxel alone did not increase the pCR rates (29.7% and 31.8%, resp., versus 32.7%; *P* = 0.69). Similarly, the GeparTrio study reported by Huober et al. showed a pCR rate of 39% in the TNBC patient subset on this trial which sought to determine the effect on pCR of switching neoadjuvant chemotherapy depending on mid-course response [20]. Patients received 2 cycles of docetaxel, doxorubicin, and cyclophosphamide (TAC), followed by either 4 or 6 more cycles of TAC in responders or 4 cycles of TAC versus capecitabine plus vinorelbine in nonresponders. In nonresponders, pCR rates were less than 10% in both treatment groups, suggesting diminished benefits of chemotherapy and lack of benefit for these particular experiment agents.

 As sporadic TNBC has clinical and molecular similarities to BRCA-1-associated breast cancers, there has been significant interest in using platinum compounds in TNBC. BRCA-1-associated breast cancers are sensitive to these agents since double-strand DNA breaks cannot be correctly repaired in cells deficient in homologous recombination repair mechanisms. Several neoadjuvant trials have therefore evaluated platinum agents in TNBC patients (Table 2). Alba et al. investigated whether the addition of carboplatin to standard chemotherapy in patient with TNBC would lead to an increase in the pCR rates in the neoadjuvant setting [21]. Patients received epirubicin plus cyclophosphamide (EC) followed either by docetaxel or docetaxel plus carboplatin. The addition of the platinum did not improve pCR rates (35% versus 30%) in this study. Silver et al. evaluated the efficacy of neoadjuvant cisplatin in 28 patients with TNBC [22]. All patients received 4 cycles of cisplatin preoperatively, followed by definitive surgery, and then adjuvant chemotherapy and/or radiation as per their treating physicians. The pCR rate was 21% (6 of 28 patients), while 64% (18 of 28) achieved either a clinical complete or partial response. The efficacy of neoadjuvant cisplatin in TNBC versus non-TNBC was also compared in a small retrospective study by Sirohi et al. [23]. Complete response rates by clinical exam were higher for those with TNBC (88%) versus the non-TNBC group (51%). Paradoxically survival outcomes were worse for the TNBC group despite higher rates of initial response to chemotherapy. This has also been seen in a study which showed that, despite a higher rate of chemosensitivity, patients with TNBC had a worse outcome that those with ER positive disease [29]. Multiple other small studies have also evaluated neoadjuvant platinum-based therapy in patients with TNBC with varying results [18, 24, 25, 30–34]. While current data provides insufficient evidence for the routine use of platinum-based therapy in patients with TNBC, several ongoing prospective studies will help define the role of these agents in the treatment of this subset of patients. Notable among these studies the GeparSixto study exploring the addition of carboplatin to neoadjuvant therapy for patients with early-stage TNBC as well as HER2-positive disease [26] demonstrated an improvement in pCR rate from 37.9% to 58.7% (*P* < 0.05) with the addition of carboplatin in the TNBC group but not in the HER2+ breast cancer group (33.1% with carboplatin and 36.3% without carboplatin, n.s.) (ASCO 2013, abstract 1004). The Cancer and Leukemia Group B (CALGB) 40603 is evaluating the addition of carboplatin and/or bevacizumab to standard chemotherapy in the neoadjuvant setting for TNBC [26]. The challenge remains to identify the subpopulation with DNA repair defects that render them particularly platinum sensitive. 

## 4. Neoadjuvant Targeted Therapy for TNBC

Different biologics such as antiangiogenic agents, poly-ADP ribose polymerase (PARP) inhibitors, and other small molecule inhibitors are being evaluated in patients with TNBC. As a higher level of vascular endothelial growth factor (VEGF) has been detected in TNBC [35], the VEGF inhibitor, bevacizumab, has been investigated in patients with TNBC. Two large randomized clinical trials have been recently reported. The GeparQuinto trial was designed to evaluate different neoadjuvant approaches in patients with HER2-negative breast cancers, HER2-negative cancers that did not have a response to initial neoadjuvant chemotherapy, and HER2-positive breast cancers [36]. The primary goal of the HER2-negative portion of this trial was to compare pCR rates with and without bevacizumab added to chemotherapy. All patients received EC followed by docetaxel. Patients were randomized to receive bevacizumab or no additional therapy. Those who did not respond to EC were then randomly assigned to paclitaxel with or without everolimus. Among 663 patients with TNBC, the pCR rates were 27.9% in the no bevacizumab group and 39.3% in the group that received chemotherapy plus bevacizumab (*P* = 0.003). These findings were not confirmed in the other large neoadjuvant study, NSABP B-40 [19]. Here, although there was a numerically higher pCR rate in the patients with TNBC that received bevacizumab, this was not significant. In contrast to the previous study, patients with hormone receptor (HR) positive disease who received bevacizumab had a higher pCR rate than those who did not receive it. Bevacizumab plus chemotherapy led to an increase in toxicity in both clinical trials, most notably hypertension. Given these differences, it is premature to speculate that adding bevacizumab to chemotherapy will benefit all patients with TNBC. CALBG 40603 has completed accrual and will hopefully shed light on which subset of TNBC, if any, will benefit from bevacizumab.

 PARP enzymes mediate repair of single-strand DNA breaks through base excision repair mechanism. Loss of PARP enzymes will therefore lead to accumulation of single-strand breaks which under normal circumstances ought to be repaired by the homologous recombination pathway. Since the homologous recombination pathway is deficient in BRCA-associated breast cancers, PARP inhibition leads to “synthetic lethality” in these cancers. As TNBC have clinicopathologic similarities to BRCA associated breast cancers, PARP inhibitors are attractive treatments for this group of patients. Olaparib has been investigated in patients with advanced TNBC- or BRCA-associated breast cancer with mixed results [37–39]. Other neoadjuvant studies are ongoing or recently reported. These include the SOLTI NEOPARP [27], which investigates iniparib, a drug initially thought to be a PARP inhibitor which was later disproven, plus paclitaxel versus, paclitaxel alone as neoadjuvant treatment in TNBC patients and I-SPY 2, which employs an adaptive trial design. A subset of TNBC patients on this trial will receive paclitaxel with or without veliparib in the neoadjuvant setting [26]. 

Other features of TNBC such as activation of the PI3 K/AKT/mTOR pathway and notch signaling and overexpression of EGFR and c-KIT continue to be exploited for treatment opportunities [26, 28] (Table 2).

## 5. Pathologic Complete Response (pCR)

Achievement of pCR is highly predictive of long-term outcome for patients with TNBC despite the various definitions of pCR applied in individual studies [29, 40–42], including ypT0 ypN0 (no invasive or noninvasive residual in breast or nodes) [43], ypT0/is ypN0 (no invasive residual in breast or nodes, noninvasive breast residual allowed) [44–46], ypT0/is yN0/+ (no invasive residual in the breast, noninvasive breast residuals and infiltrated lymph nodes allowed) [2, 47], and ypT≤1mic ypN0/+ (no gross invasive residuals in the breast, focal invasive and noninvasive residuals in breast and infiltrated lymph nodes allowed) [48]. However, the most stringent definition of pCR, no invasive and in situ (DCIS) residuals in breast and nodes, has been suggested to be most prognostic by a recent large pooled analysis of 6,377 breast cancer patients enrolled in seven prospective clinical trials of neoadjuvant chemotherapy [42]. The poorer prognosis associated with residual DCIS, in the absence of invasive cancer in either breast or nodes, was surprising since theoretically most patients with DCIS are cured therefore suggesting that these patients may harbor occult invasive cells that are hard to identify or the residual DCIS following chemotherapy is somehow biologically different. However, an earlier retrospective analysis of 2,302 patients who received neoadjuvant chemotherapy at a single institution showed no difference in 5-year or 10-year DFS between groups with ypT0 ypN0 and ypTis pN0 [49], although the sample size was smaller. In practice, both ypT0 ypN0 and ypT0/is ypN0 commonly used to indicate pCR than ypT0/is yN0/+ and ypT≤1mic ypN0/+. 

TNBC is associated with a significantly higher rate of pCR to standard neoadjuvant compared to ER+ HER2− disease. The rate of pCR approximates 20% or higher for TNBC in various studies [29, 40, 41]. While those who achieved pCR have an excellent long-term outcome, the majority of patients with TNBC do not achieve pCR and suffer a dramatically worse outcome compared to those with ER+ disease [29, 41]. The recurrence rate for those who did not achieve pCR is as high as 40–50% at 5 years for TNBC [41, 42], explaining the paradox of worse clinical outcome in general despite the higher likelihood of chemosensitivity. There is a significant unmet clinical need for novel therapeutics development in the resistant population. 

In addition to pCR, quantitation of residual disease burden in the breast and lymph nodes, including the use of residual cancer burden (RCB) [50] and Miller-Payne Scores [51], could further categorize those with non-pCR into separate prognostic groups. Although these scorings are not routinely assessed in clinical practice, some have been incorporated in clinical trials to identify the highest risk population for novel therapeutics development in the adjuvant setting. One example is the ongoing study conducted by the Hoosier Oncology Group that assesses PARP inhibition in patients with triple negative breast cancer or ER/PR+, HER2 negative disease with known BRCA1/2 mutations (NCT01074970) who had Miller-Payne response in the breast of 0–25 or RCB classification of II or III following neoadjuvant chemotherapy. 

## 6. The Neoadjuvant Setting as a Research Platform

The induction of a pCR in response to neoadjuvant chemotherapy effectively categorizes heterogeneous TNBC into high- and low-risk groups. Unfortunately, there has not been any effective therapy for those with chemotherapy-resistant disease. There has been significant interest in analyzing residual tumors in order to uncover molecular aberrations as potential therapeutic targets. Balko et al. presented an analysis with targeted next-generation sequencing (NGS) of 182 oncogenes and tumor suppressors and gene expression profiling of residual breast cancer after neoadjuvant chemotherapy in 102 patients with TNBC in the recent San Antonio Breast Cancer Symposium [52]. Eighty-nine posttreatment tumors were evaluable for gene expression and included basal-like (64%), HER2-enriched (19%), luminal A (6%), luminal B (6%), and normal-like (5%) subtypes. Of 81 tumors evaluated by NGS, common genetic aberrations include mutations in TP53 (89%), MCL1-amplification (27%), and MYC-amplification (21%). Common pathway alterations at the DNA level included PI3 K/mTOR pathway (33%), cell cycle genes (31%), DNA repair pathway (20%), and the Ras/MAPK pathway (12%). Sporadic growth factor receptor amplifications occurred. MYC amplification was an independent poor prognostic indicator of recurrence-free survival (RFS) and overall survival (OS). Adjuvant trials are being designed to address specific hypothesis originated from this investigation. This study underscores the complexity and the diversity of genetic abnormalities in the residual tumors that we are up against. Effective preclinical models and novel trial designs are needed in order to decipher the driver genetic events and validate potentially actionable targets for TNBC. 

Improvement in the rate of pCR has been considered an accepted endpoint for neoadjuvant clinical trials in patients with TNBC so that drugs could be tested efficiently. The ability to obtain sufficient high-quality tumor material before and after therapy makes it possible to correlate biomarkers with pathologic response. Furthermore, specific biomarkers could be incorporated in the trial design as an eligibility criteria or stratification factor to enhance the power to address specific biomarker hypothesis. 

To facilitate early access of potentially active drugs for patients with high-risk disease, the Food and Drug Administration (FDA) has recently outlined a pathway for accelerated drug approval based on pCR from neoadjuvant trials. However, disease-free survival (DFS) and overall survival (OS) remain the standard endpoints for eventual drug approval. Large neoadjuvant trials that are powered for these endpoints may be preferred. Alternatively, adjuvant trials could be conducted while the drug receives accelerated approval so that the DFS and OS endpoints could be assessed.

## 7. Controversies and Unanswered Questions

The data currently available on neoadjuvant chemotherapy in primary breast cancers leaves many questions unanswered. For instance, what is the role of further non cross-resistant systemic therapy in TNBC patients who do not respond to initial neoadjuvant chemotherapy? In the aforementioned GeparTrio study [20, 53], patients who did not respond initially to 2 cycles of TAC were randomized to further chemotherapy with TAC or capecitabine plus vinorelbine. pCR rates seen in nonresponders who received further cycles of TAC were 7.3% versus 3.1% in the capecitabine plus vinorelbine group supporting the hypothesis that chemotherapy-resistant patients may not benefit from a switch to different conventional chemotherapy agents/regimens. Similarly, the Aberdeen trial reported a pCR rate of 2% in patients who received neoadjuvant docetaxel after proving unresponsive to a doxorubicin-based neoadjuvant regimen, while those who were initially chemosensitive to the doxorubicin-containing regimen and received further docetaxel had a pCR of 31% [54]. Although neither study was restricted to patients with TNBC, one can assume this to hold true in that subtype as well. Trials are ongoing to address this specific issue in patients with TNBC [26].

At the other end of the response spectrum questions are beginning to be asked as to whether a subset of TNBCs who achieve pCR do not require the full complement of local treatments (completion node dissection and/or extensive locoregional radiation). The consideration to withhold definitive surgery from any patient who achieves such a clinical or radiological response to neoadjuvant chemotherapy is certainly premature at this time considering that our current tools for assessing response to neoadjuvant chemotherapy are very insensitive to microscopic but clinically relevant residual disease. A small European study compared the accuracy of clinical examination, mammography, ultrasonography, and dynamic MRI in predicting response to neoadjuvant chemotherapy [55]. All fifteen patients underwent definitive surgery and had their pathology results compared with clinical examination and radiologic findings during and after neoadjuvant chemotherapy. MRI performed the best in discriminating between the presence and absence of residual disease in 100% of cases and was also able to evaluate the extent of residual disease in 80%. Mammography was able to do this in 86% and 53%, respectively. However, MRI was only able to detect multifocal/multicentric disease in 67% following neoadjuvant chemotherapy. As the pattern of residual disease on histologic examination following neoadjuvant chemotherapy may vary, MRI or other imaging modalities may therefore not accurately detect small volume residual disease, particularly if located in different quadrants. Another larger study compared clinical examination with ultrasonography and mammography [56]. Tumor size estimation by clinical examination and then by both imaging techniques did not correlate well with pathologic results. PET/CT scanning as a modality to predict response to neoadjuvant therapy in TNBC was recently reported by Groheux et al. [57]. pCR was found in 6 of 20 (30%) study patients. The area under the receiver operating characteristic curve representing the ability of pre- to postneoadjuvant chemotherapy change in SUV in predicting pathology findings of non-pCR versus pCR was 0.881. Imaging research continues to advance as tools are refined; the question as to which group of patients may safely omit surgery may eventually need to be addressed. 

Another key question concerns the best timing of sentinel lymph nodal biopsy (SLNB) in patients undergoing neoadjuvant chemotherapy. The benefits and disadvantages of performing this procedure either before or after chemotherapy are summarized in Table 3. The strongest argument in favor of performing this before chemotherapy is that it accurately measures the presence or absence of nodal disease for proper staging considering that a number of patients will have their nodal disease treated by chemotherapy. On the other hand, SLNB done before surgery could affect the assessment of pathologic response in lymph node. In addition, SLNB done after preoperative chemotherapy is successful [58] and may decrease morbidity by eliminating multiple procedures or perhaps even a full axillary dissection. In patients with histologically confirmed positive lymph nodes, the accuracy of SLNB after neoadjuvant chemotherapy is the subject of ongoing trials [26]. Theoretically when chemotherapy successfully clears lymph-nodes, completion dissection and regional nodal radiation are unnecessary. 

## 8. Conclusion 

 Neoadjuvant chemotherapy is now a standard of care for a subgroup of patients with TNBC, particularly those with clinical stage 2B or 3 disease, but it could be reasonably argued that any patient with a biopsy that shows invasive disease would be eligible. This approach promotes breast conservation and provides an important platform for translational research. pCR is an appropriate surrogate for measuring long-term clinical outcome in TNBC. Although pCR rates to standard chemotherapy are higher in TNBC compared to HR positive breast cancer, the large proportion of patients with TNBC that do not achieve pCR presents a therapeutic challenge. Further studies are therefore critical to identify predictors of resistant population so that mechanism-based interventions could be designed in the neoadjuvant setting to improve pCR rates and long-term outcome. Studies are also needed to address ongoing controversies and debates including the use of axillary surgery and the role of postoperative regional radiotherapy (nodes and chest wall in the setting of mastectomy) in patients with pCR.

## Figures and Tables

**Table 1:** Genomic and proteomic features of basal-like breast cancer (data from TCGA [7]).

Pathways and analysis	Aberrations
TP53 pathway	TP53 mut (84%); gain of MDM2 (14%)
PIK3CA/PTEN pathway	PIK3CA mut (7%); PTEN mut/loss (35%); INPP4B loss (30%)
RB1 pathway	RB1 mut/loss (20%); cyclin E1 amp (9%); high expression of CDKN2A; low expression of RB1
Copy number	Most aneuploid; high genomic instability; 1q, 10p gain; 8p, 5q loss; MYC focal gain (40%)
Proteomic analysis by reverse phase protein array	High expression of DNA repair proteins, PTEN and INPP4B loss signature (pAKT)

**Table 2:** Summary of completed and ongoing studies of neoadjuvant chemotherapy and targeted therapy in TNBC.

Study	*N*	Treatment	Primary endpoint
Platinum agents			
Alba et al. [21]	94	EC^a^ followed by D^b^ versus EC followed by D plus Cb^c^	pCR^h^
Silver et al. [22]	28	Cisplatin	pCR
Sirohi et al. [23]	62	Cisplatin	Clinical response rates, OS^i^
Kern et al. [24]	27	Carboplatin plus docetaxel	pCR
Tiley et al. [25]	12	AC^d^ followed by P^e^ with Cb	pCR
GeparSixto [26]	Ongoing	Carboplatin plus standard chemotherapy	pCR

Targeting angiogenesis			
Ryan et al. [18]	51	Cisplatin plus bevacizumab	Clinical response
CALGB 40603 [26]	Ongoing	Carboplatin and/or bevacizumab	pCR
NCT00887575 [26]	Ongoing	Sunitinib plus pactlitaxel plus carboplatin	MTD^j^, pCR
NCT01194869 [26]	Ongoing	Sorafenib plus pactlitaxel plus cisplatin	pCR

Targeting DNA damage repair			
Llombart et al. [27]	141	Iniparib plus paclitaxel	pCR
NCT00813956 [26]	Ongoing	Gemcitabine plus Cb plus iniparib	pCR
I-SPY 2 [26]	Ongoing	Veliparib plus paclitaxel	pCR

Targeting EGFR			
ICE [26]	Ongoing	Ixabepilone plus cetuximab	pCR
NCT00491816 [26]	Ongoing	Erlotinib plus chemotherapy	pCR

Targets HER3			
NCT01421472	Ongoing	Paclitaxel plus MM-121 (targets HER3)	pCR

Targets inhibitors of apoptosis (IAP)			
NCT01617668	Ongoing	Paclitaxel plus LCL161	pCR

Targets gamma-secretase/notch signaling pathway			
NCT01238133	Ongoing	Paclitaxel plus Cb plus RO4929097	MTD

Targets PI3K/mTOR			
Gonzalez-Angulo et al. [28]	62	T-FEC^f^ versus TR-FEC^g^	Clinical response rate at 12 weeks
NCT00930930	Ongoing	Cisplatin plus paclitaxel ± everolimus	pCR

Targets multiple tyrosine kinases			
NCT00817531	Completed	Dasatinib	Clinical response by RECIST

^a^Epirubicin cyclophosphamide, ^b^docetaxel, ^c^carboplatin, ^d^doxorubicin cyclophosphamide, ^e^paclitaxel, ^f^paclitaxel 5-flourouracil epirubicin cyclophosphamide, ^g^paclitaxel everolimus 5-flourouracil epirubicin cyclophosphamide, ^h^pathologic complete response, ^i^overall survival, ^j^maximum tolerated dose.

**Table 3:** Benefits of performing SLNB before or after neoadjuvant chemotherapy.

Benefits of SLNB before neoadjuvant chemotherapy	Benefits of SLNB after neoadjuvant chemotherapy
Allows for accurate lymph node staging	May eliminate the need for two surgical procedures, thus decreasing morbidity
May impact on choice of systemic therapy and radiation therapy	If clinically positive lymph nodes are down staged, patients may avoid a full axillary dissection
Accuracy of SLNB may be superior by avoiding lymphatic changes induced by chemotherapy	Administration of systemic therapy is not delayed
